# Regeneration in European beech forests after drought: the effects of microclimate, deadwood and browsing

**DOI:** 10.1007/s10342-022-01520-1

**Published:** 2022-12-08

**Authors:** Dominik Thom, Christian Ammer, Peter Annighöfer, Réka Aszalós, Sebastian Dittrich, Jonas Hagge, William S. Keeton, Bence Kovacs, Ole Krautkrämer, Jörg Müller, Goddert von Oheimb, Rupert Seidl

**Affiliations:** 1 Ecosystem Dynamics and Forest Management Group, School of Life Sciences, Technical University of Munich, Hans-Carl-Von-Carlowitz-Platz 2, 85354 Freising, Germany; 2 Gund Institute for Environment, University of Vermont, 617 Main Street, Burlington, VT 05405 USA; 3 Silviculture and Forest Ecology of the Temperate Zones, Faculty of Forest Sciences, University of Göttingen, Büsgenweg 1, 37077 Göttingen, Germany; 4 Forestry and Agroforestry Systems Group, School of Life Sciences, Technical University of Munich, Hans-Carl-Von-Carlowitz-Platz 2, 85354 Freising, Germany; 5 Centre for Ecological Research, Institute of Ecology and Botany, Alkotmány Út 2-4, Vácrátót, 2163 Hungary; 6 Institute of General Ecology and Environmental Protection, Department of Forest Sciences, Technische Universität Dresden, Pienner Straße 7, 01737 Tharandt, Germany; 7 Forest Nature Conservation, Northwest German Forest Research Institute, Prof.-Oelkers-Str. 6, 34346 Hann. Münden, Germany; 8 Forest Nature Conservation, Faculty of Forest Sciences, Georg-August-University Göttingen, Büsgenweg 3, 37077 Göttingen, Germany; 9 Rubenstein School of Environment and Natural Resources, University of Vermont, Burlington, VT 05405 USA; 10 Animal Ecology, Department of Ecology, Faculty of Biology, Philipps-University of Marburg, Karl-Von-Frisch-Straße 8, 35043 Marburg, Germany; 11 LPV, Thüringer Wald E. V., Rennsteigstraße 18, 98673 Eisfeld, OT Friedrichshöhe Germany; 12 Ecological Field Station Fabrikschleichach, Department of Animal Ecology and Tropical Biology, University of Würzburg, Glashüttenstraße 5, 96181 Rauhenebrach, Germany; 13 Bavarian Forest National Park, Freyunger Strasse 2, 94481 Grafenau, Germany; 14 Berchtesgaden National Park, Doktorberg 6, 83471 Berchtesgaden, Germany

**Keywords:** Seedlings, Species diversity, Structural diversity, Tree establishment, Understory

## Abstract

With progressing climate change, increasing weather extremes will endanger tree regeneration. Canopy openings provide light for tree establishment, but also reduce the microclimatic buffering effect of forests. Thus, disturbances can have both positive and negative impacts on tree regeneration. In 2015, three years before an extreme drought episode hit Central Europe, we established a manipulation experiment with a factorial block design in European beech (*Fagus sylvatica* L.)-dominated forests. At five sites located in southeastern Germany, we conducted three censuses of tree regeneration after implementing two different canopy disturbances (aggregated and distributed canopy openings), and four deadwood treatments (retaining downed, standing, downed + standing deadwood and removing all deadwood), as well as in one untreated control plot. In addition, we measured understory light levels and recorded local air temperature and humidity over five years. We (i) tested the effects of experimental disturbance and deadwood treatments on regeneration and (ii) identified the drivers of regeneration density as well as seedling species and structural diversity. Regeneration density increased over time. Aggregated canopy openings supported species and structural diversity, but reduced regeneration density. Tree regeneration was positively associated with understory light levels, while maximum vapor pressure deficit influenced tree regeneration negatively. Deadwood and browsing impacts on regeneration varied and were inconclusive. Our study indicates that despite the drought episode regeneration in beech-dominated forests persisted under moderately disturbed canopies. However, the positive effect of increased light availability on tree regeneration might have been offset by harsher microclimate after canopies have been disturbed.

**Supplementary Information:**

The online version contains supplementary material available at 10.1007/s10342-022-01520-1.

## Introduction

Regeneration is a crucial determinant for the future composition and structure of forests (Ammer et al. 2008; Donato et al. 2012; Fischer and Fischer 2012). Regeneration dynamics strongly influences habitat quality for forest-dwelling species, affects ecosystem services supply, and determines the adaptive capacity of forests to environmental change (Duveneck and Scheller 2015; Aquilué et al. 2021). The intensity and frequency of heat and drought events have increased in Central Europe (Büntgen et al. 2021) and will likely further increase in response to climate change (Seidl et al. 2017). This may challenge the survival of tree regeneration and cause a thermophilization (i.e., a decline of cold-adapted species) of the understory (Feeley et al. 2020; Caron et al. 2021). From 2018 to 2020, large parts of Central Europe experienced the most intense drought episode on record (Hari et al. 2020; Pińskwar et al. 2020). Tree mortality was widespread and unprecedented in at least the past 170 years (Senf and Seidl 2021). Large-scale mortality also occurred in tree species that were expected to tolerate the climatic change expected for the coming decades, such as European beech (*Fagus sylvatica* L.) (Schuldt et al. 2020). In addition, drought triggered a large wave of tree mortality from other agents such as bark beetles (Hlásny et al. 2021).

Forest disturbances alter microclimatic conditions, particularly increasing light availability in gaps as well as local temperatures during the growing season while reducing humidity (Kovács et al. 2020; Thom et al. 2020). Alterations of understory microclimate caused by canopy openings are most pronounced close to the forest floor (Blumröder et al. 2021). Thus, seedlings are particularly exposed to changing microclimatic conditions. In addition, as the roots of seedlings are short and their storage capacity is low, they are more sensitive to drought conditions than canopy trees (E Silva et al. 2012; Leuschner 2020). Hence, as climate change intensifies and changing disturbance regimes increasingly reduce the microclimatic buffering capacity of the forest canopy (i.e., the capacity to dampen fluctuations in temperature and air humidity (De Frenne et al. 2021)), regeneration success (i.e., the density and diversity of tree regeneration) could be threatened (Miller and McGill 2019; Rammer et al. 2021). Regeneration failure constitutes a great challenge to forestry as regime shifts (e.g., from forest to shrubland) or the loss of important tree species likely have negative consequences for ecosystem services and biodiversity (Barnosky et al. 2012; Reyer et al. 2015). In contrast, canopy disturbances can be important drivers of tree regeneration by increasing the availability of light for photosynthesis (Brüllhardt et al. 2021) and by reducing competition for water between overstory and understory trees (Petriţan et al. 2011). In particular, medium-sized gaps created by disturbance increase the heterogeneity in conditions close to the forest floor, favoring diversity in tree regeneration (Helbach et al. 2022). Overall, it remains uncertain how different patterns of disturbance (e.g., gap sizes and gap structures) affect regeneration success, in particular, when considering increasing climatic extremes.

Another threat to the successful regeneration of trees is browsing. Overabundant wild ungulate populations cause regeneration failure and socio-ecological conflicts in many parts of Europe (Valente et al. 2020). Browsing pressure from ungulates, such as roe deer (*Capreolus capreolus* L.) and red deer (*Cervus elaphus* L.), is considerably elevated compared to natural conditions in parts of Central Europe as a consequence of missing predators, land use (i.e., highly fragmented landscapes providing high energy forage on agricultural lands), and winter feeding of wild ungulates (Schulze et al. 2014; Valente et al. 2020). In addition, browsing pressure is strongly contingent on wildlife management strategies and site conditions (Hothorn and Müller 2010; Heurich et al. 2015). As some ungulates exhibit preferential browsing behavior, they alter interspecific competition among tree regeneration (Boulanger et al. 2009; Ohse et al. 2017). For instance, in Central Europe roe deer generally prefers sycamore maple (*Acer pseudoplatanus* [L.]) and silver fir (*Abies alba* [Mill.]) over European beech and Norway spruce (*Picea abies* [Karst.]) (Ohse et al. 2017; Szwagrzyk et al. 2020).

Enhancing the availability of deadwood has been suggested to foster tree regeneration in multiple ways. Downed deadwood has been shown to protect tree regeneration from ungulates as it serves as a physical barrier and hides seedlings (Hagge et al. 2019). In addition, some tree species, such as Norway spruce and silver fir, have specialized seeds adapted to germinate on downed deadwood. Germinating on such “nurse logs” gives them a head start compared to seedlings germinating on the forest floor, e.g. in terms of outgrowing competing understory vegetation. Nurse logs also raise seedlings above late spring snow cover and thus extend the growing season for regenerating trees (de Andrés et al. 2014). Moreover, deadwood ameliorates microsite conditions by regulating local temperatures and storing and releasing water and nutrients, potentially supporting the growth of regenerating tree cohorts (Bonetti et al. 2021; Marangon et al. 2022).

To date, there is only limited understanding of the tree regeneration response toward disturbance-induced alterations of the microclimate during extreme weather events. In autumn of 2015 (i.e., three years before the region was affected by two extremely dry and hot years in 2018 and 2019, see Figs. S1 and S2), a new manipulation experiment (“BioHolz”) was established in Bavaria, Germany. In a factorial replicated block design different disturbance and deadwood treatments were implemented, allowing for a standardized assessment of regeneration success. In the present study, we analyzed the outcome of the BioHolz experiment by (i) testing the effects of disturbance and deadwood treatments on tree regeneration and (ii) quantifying the drivers of regeneration density as well as species and structural diversity of tree regeneration. Given that regeneration in the European beech forests studied here is frequently light-limited, we expected the highest regeneration density in gaps with high light availability (H1). We also assumed the highest tree species diversity in the regeneration layer in distinct gaps (i.e., aggregated removal of trees), where light and soil moisture conditions are suitable for a larger range of species compared to scattered single-tree mortality (i.e., dispersed removal of trees) (Helbach et al. 2022) (H2). Moreover, we expected that the heterogeneous light conditions created by an aggregated removal of trees would increase structural diversity, as some individuals receive more light than others and thus develop faster (H3). As an alternative hypothesis to H1 and H2, we tested potential negative effects of canopy openings on density and diversity of seedlings due to a reduction of microclimatic buffering of the pronounced heat and drought conditions during the summers of 2018 and 2019 (H4). The regeneration of late-successional species, such as European beech, is expected to be more sensitive toward heat and drought extremes compared to early- or mid-seral species such as Scots pine (*Pinus sylvestris* [L.]) and oak species (*Quercus* spp.) (van Hees 1997). Regeneration of European beech could thus be particularly dependent on microclimatic buffering by a forest canopy (Vodde et al. 2011; Vilhar et al. 2015). Next, we expected browsing to reduce regeneration density, species diversity, and structural diversity (H5). Lastly, we hypothesized a positive effect of deadwood on all indicators of regeneration, as deadwood enhances water and nutrient availability and shelters seedlings from ungulates (H6).

## Materials and methods

### Study area

**Fig. 1:**
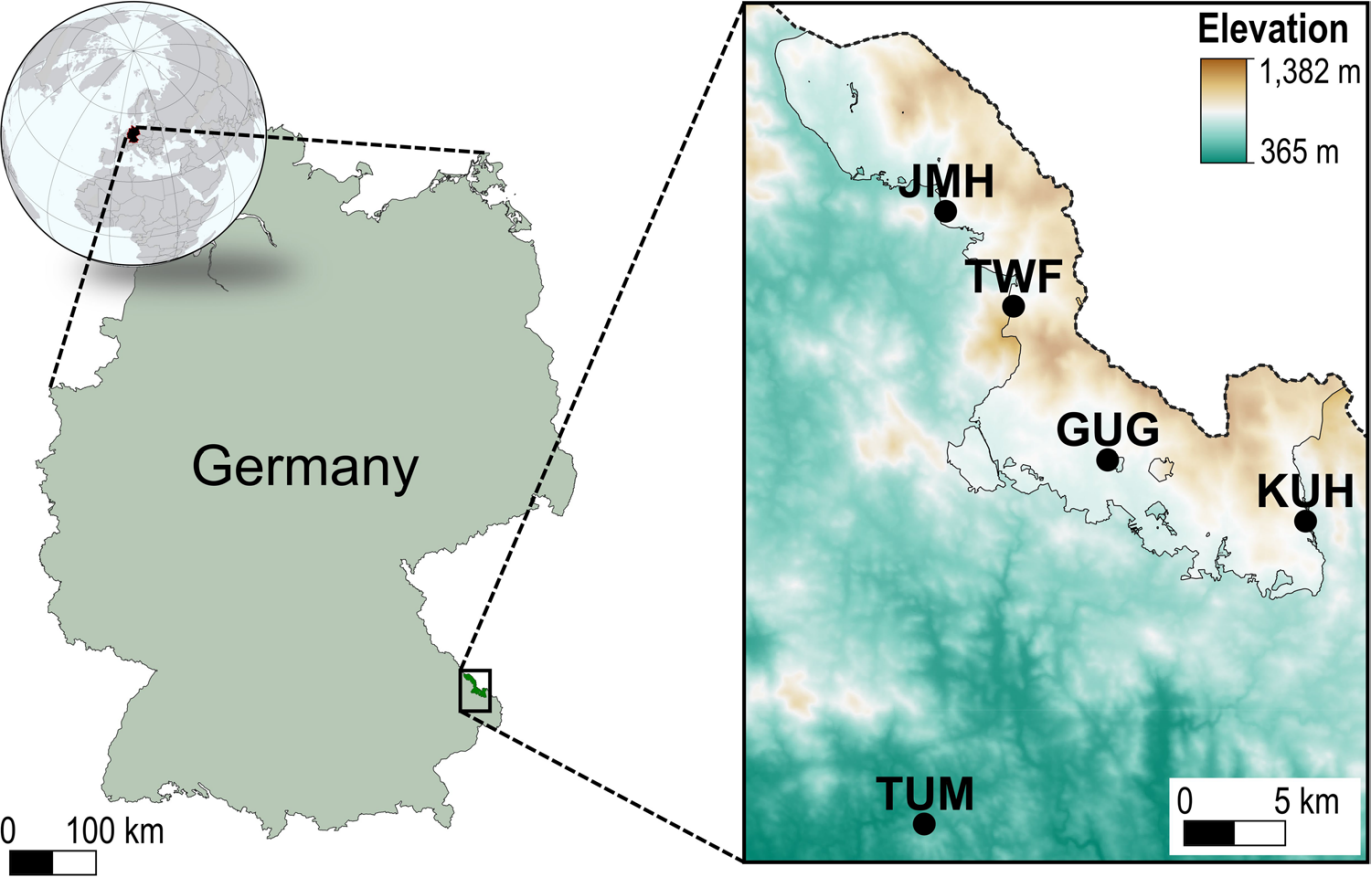
Study location and experimental sites. Borders of the Bavarian Forest National Park are highlighted

Our manipulation experiment is located in southeastern Germany (Fig. 1). The study area comprises four sites in the Bavarian Forest National Park including Guglöd (GUG), Jungmaierhütte (JMH), Kuhhüttenberg (KUH), and Trinkwasserspeicher Frauenau (TWF), as well as one site in the close proximity of the National Park in Thurmansbang (TUM). All sites are located in sub-montane–montane elevation zones characterized by a sub-Atlantic climate as well as moderately podzolic Cambisols over gneiss and granite parent material. Prior to implementing experimental treatments, all forests were in a mature development stage dominated by European beech admixed with Norway spruce and other tree species (Table 1). Stands originated from shelterwood-cutting and were, therefore, characterized by a single canopy layer and low variation in tree age and size prior to the experiment. Management ceased about 50 years ago, and forests have not noticeably been affected by natural disturbance in recent decades.

**Table 1:** Site topography and forest attributes (modified from Thom et al. 2020)

Category	Attribute	Unit	Location				
			GUG	JMH	KUH	TUM	TWF
Coordinates		UTM	32 U 824,074 5,429,269	32 U 815,223 5,440,885	32 U 834,390 5,426,884	32 U 816,421 5,410,171	32 U 819,000 5,436,191
Topography	Elevation	m	837	857	852	483	1055
	Slope	degree	7.1	13.0	18.1	10.9	11.0
	Aspect	dim	W	N	E	N	NE
Forest structure	Live basal area	m^2^ ha^−1^	32.4 (5.6)	30.9 (5.3)	34.3 (5.5)	31.6 (4.0)	41.4 (4.5)
	Stand density	n ha^−1^	540 (164)	418 (159)	388 (89)	443 (89)	864 (173)
	Mean DBH	cm	24.3 (5.2)	29.1 (4.9)	30.7 (2.5)	26.3 (3.6)	23.4 (2.3)
	Mean tree height	m	26.1 (2.5)	29.4 (3.9)	28.3 (1.7)	25.9 (2.0)	21.9 (3.1)
	Standing deadwood basal area	m^2^ ha^−1^	3.3 (3.4)	3.4 (3.1)	2.9 (3.0)	3.1 (2.5)	4.0 (3.4)
	Downed deadwood volume	m^3^ ha^−1^	21.8 (27.2)	30.1 (36.7)	27.0 (32.2)	19.8 (20.8)	23.6 (30.9)
Composition	Proportion of European beech on total basal area	%	83.4 (14.0)	92.0 (3.1)	70.4 (18.1)	77.5 (16.7)	81.2 (8.1)
	Proportion of Norway spruce on total basal area	%	12.3 (11.8)	7.1 (3.0)	27.8 (19.4)	20.4 (15.1)	17.6 (8.9)
	Proportion of other tree species on total basal area	%	4.4 (5.4)	0.9 (1.1)	1.8 (2.4)	2.0 (3.1)	1.3 (2.4)
	Effective number of canopy tree species (exponent of Shannon index)	n	1.6 (1.3)	1.3 (1.1)	1.8 (1.2)	1.6 (1.3)	1.6 (1.1)

Inventories were performed in 2016 a few months after the implementation of experimental treatments. Presented are means and standard deviations (in parentheses) across all nine plots (i.e., eight treated plots and one untreated control plot) per site. The effective number of canopy tree species is expressed as the exponent of the Shannon index and is calculated based on basal area of live trees. DBH = diameter at breast height

### Experimental design

**Fig. 2:**
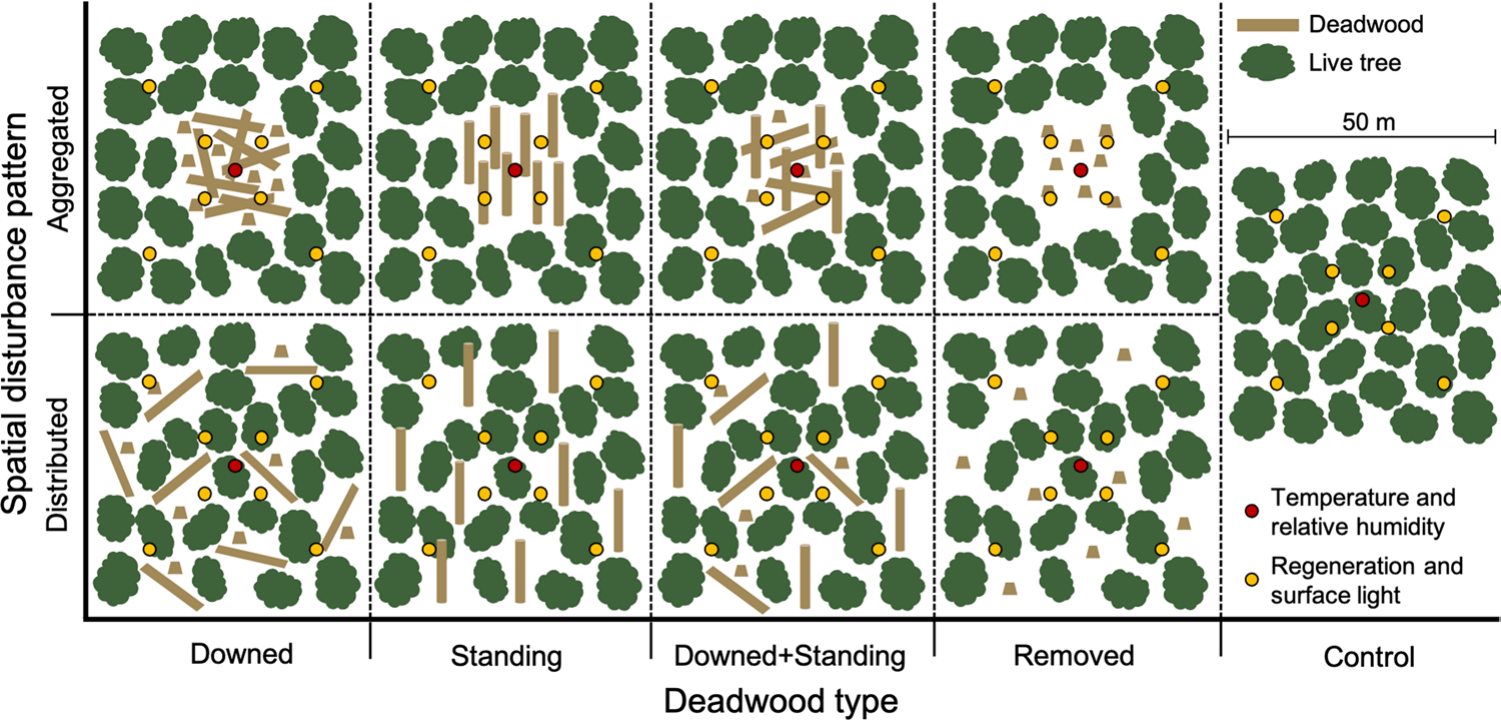
Experimental design. The factorial replicated block design includes two spatial patterns of canopy disturbance (i.e., aggregated and distributed removal of trees) and four different deadwood treatments as well as an untreated control for each of the five experimental sites (i.e., 45 plots in total). In all treatments, disturbance severity was kept constant at ~ 25% basal area removed. Recording positions of temperature and relative humidity as well as of regeneration and understory light conditions are shown as red and orange points, respectively

**Fig. 3:**
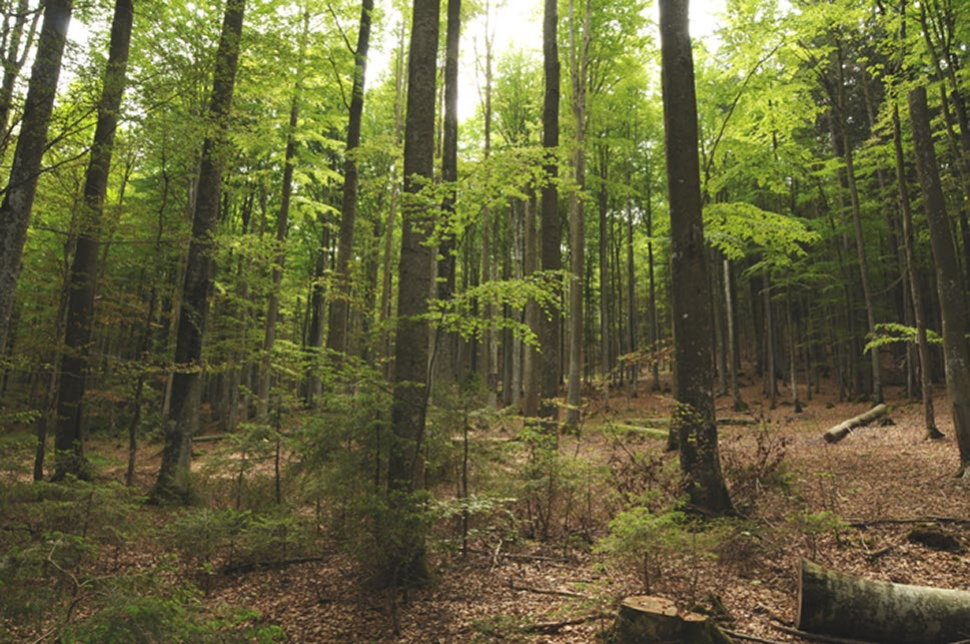
An example of tree regeneration on a plot with the experimental treatment distributed canopy disturbance with retention of both standing and downed deadwood. The photograph was taken 2017 in Jungmaierhütte (JMH)

Manipulations in our experiment followed a factorial block design (Fig. 2). At the five experimental sites, eight 2,500-m^2^ plots were treated by removing approximately 25% of live basal area. Two different patterns of canopy disturbance were created, removing trees randomly across the plot or creating one contiguous gap of 25 m × 25 m (625 m^2^) around the plot center. The deadwood produced by these canopy disturbances was either retained as downed logs, standing dead trees, both downed and standing deadwood, or was entirely removed. Standing deadwood was generated by cutting trees below the first canopy branch, on average at a height of 8.3 m. The crowns of the cut trees were removed from the plots. Because of the factorial design, treatments resulted in similar deadwood amounts across the plots of a site. In addition, each experimental site contained one untreated control plot. In total, nine plots (8 treatments + 1 control) were replicated across five sites, totaling 45 plots included in the analysis (Figs. 2, 3).

### Data collection

We installed automatic data loggers for measuring temperature and relative air humidity (model: UT 330C; UNI-Trend Technology Co. Ltd., Dongguan, CHN) at a height of 1.2 m above the ground on a pole in the center of each plot (Fig. 2). Data loggers were used to record temperature and humidity at an hourly time step in the summer months (June–August) from 2016 to 2020. All loggers were equipped with a white, passively ventilated radiation shield to protect them from direct sunlight. Drought and its influence on tree regeneration are particularly pronounced in summer (Chen et al. 1999; Baker et al. 2014; Zellweger et al. 2019). To assess the effect of the drought episode of 2018–2019 on regeneration, we thus focused on summer microclimate.

In addition to temperature and humidity, we collected data about the light regime on each plot. Light is a crucial factor limiting tree regeneration, particularly in forests of shade-tolerant species such as European beech (Brüllhardt et al. 2021). Furthermore, the light regime was found to strongly determine local temperature and air humidity in previous analyses (Ehbrecht et al. 2019; Thom et al. 2020). Light measurements were taken in 2016, 2017, and 2020. We measured light conditions with hemispherical photography (Solariscope SOL 300B, Ing.-Büro Behling, Hermannsburg, GER) at eight systematically distributed grid points around the plot center (Fig. 2). Measurements were taken in the summer months at the same height at which the automatic data loggers were installed (i.e., 1.2 m). As an indicator for understory light conditions, we derived total site factor (TSF), which combines direct (DSF) and diffuse light (ISF) into one index. DSF and ISF were not analyzed separately here as they were highly correlated (*r* = 0.67) and their correlation coefficients with TSF were both > 0.9, indicating that TSF is an integral indicator for describing the light regime at our experimental plots.

Data on tree regeneration were collected in 2016, 2018, and 2020 at eight 4-m-radius subplots (i.e., at 50.2 m^2^ per subplot and a total of 401.9 m^2^ per plot) around the plot center, corresponding to the location of light measurements (Fig. 2). We counted all live seedlings with a minimum height of 20 cm up to a diameter at breast height (1.3 m) of 6 cm. Moreover, we collected information about species, and tree height in three tree height classes (20–30 cm, 31–50 cm,  > 50 cm). No additional height classes were recorded as regeneration has not reached 1.3 m in height in the vast majority of plots. Browsing influence (i.e., whether the terminal shoot of an individual was browsed or not) was determined only in the year 2020.

### Data processing

We harmonized and cleaned the microclimate logger data by omitting days with unrealistic and incomplete measurements. Next, we obtained vapor pressure deficit (VPD) from temperature and humidity measurements by subtracting the actual pressure of water vapor in the air from saturated vapor pressure. As VPD defines the difference between saturated and actual vapor pressure, it is an indicator for the drying capacity of the air affecting plant hydraulic functioning (Ficklin and Novick 2017).

Focusing on extreme conditions for regeneration, we aggregated hourly records to maximum daily temperature (*T*_max_) and VPD (VPD_max_). We thus focused on the hottest and driest conditions observed, and their potential effect on growth (e.g., due to stomata closure) and mortality (e.g., due to drought or burned plant tissue) of regenerating trees (Keenan and Kimmins 1993; Jagadish et al. 2021). In total, we obtained 10,168 daily observations of *T*_max_ and VPD_max_. Subsequently, we aggregated *T*_max_ and VPD_max_ to the average summer maxima for each year and then averaged all years to obtain the average summer maxima between 2016 and 2020 at each plot.

Next, we determined average annual light conditions at plot level. The eight light measurements taken per plot in 2016, 2017, and 2020 were averaged by year. We used linear interpolation to derive light conditions for the years 2018 and 2019 before averaging across all years to estimate average light conditions at each plot since treatments were carried out (Fig. S3).

We analyzed three indicators of tree regeneration as response variables. First, we summed the eight subplots to derive regeneration density per year and plot. Second, we computed species diversity based on these data. Specifically, we calculated the exponent of the Shannon index (based on stem density proportions), which can be interpreted as the effective number of species in the regeneration (Jost 2006). Lastly, we derived structural diversity in the regeneration cohort per year and plot using the exponent of the Shannon index across seedling height classes. Note that the maximum number of effective height classes is three (i.e., if stems are equally distributed across all three height classes), while the effective number of tree species has no *a priory* upper bound.

### Statistical analysis

In a first step, we analyzed the overall treatment effects on our study variables. In particular, we focused on microclimatic conditions (i.e., *T*_max_, VPD_max_, and light conditions) over the period 2016 to 2020, browsing intensity (i.e., the proportion of seedlings browsed) in 2020, and tree regeneration indicators (i.e., regeneration density, species diversity, and structural diversity) in 2020 to quantify the effect of our treatments (Table S1). For a visual interpretation of treatment effects, we performed an ordination across all variables using non-metric multidimensional scaling (NMDS). We standardized all variables by z-score transformation, that is by centering (i.e., subtracting the mean) and scaling (i.e., dividing by the standard deviation), before deriving a Gower distance matrix that allows the inclusion of missing data (Brown et al. 2012). Subsequently, we fitted NMDS models with 1–6 dimensions using all variables of all 45 plots. We analyzed NMDS model performance based on screeplots of the stress value. While model performance increases with higher dimensionality, the interpretability decreases by adding dimensions. We decided to proceed with a 3-dimensional NMDS as suggested by analyzing the inflection point (the “elbow”) of the screeplot. The model’s goodness of fit was high as indicated by a stress value of 0.082, and a very high correlation between ordination distance and observed dissimilarity (non-metric fit *R*^2^ = 0.993; linear fit *R*^2^ = 0.978).

Next, we tested for significant differences among treatments using a multilevel permutation-based analysis of similarities (ANOSIM) nested by study site. The ANOSIM statistic R ranges from 0 (groups are similar) to 1 (groups are dissimilar). For a more detailed analysis of different treatment outcomes, we tested the effect of canopy disturbance and deadwood treatment for significant differences (*α* = 0.05) using pairwise independence tests with a Benjamini–Hochberg p-value adjustment (Mangiafico 2021). Pairwise independence tests are based on permutations and are neither formerly restricted by the number of observations, nor do they require a normal data distribution (Hothorn et al. 2008). Moreover, we used pairwise independence tests to compare differences in regeneration density, species diversity, and structural diversity over time. We performed additional analyses to test regeneration density changes for the two most common species and to investigate the regeneration density of trees > 50 cm over time.

Subsequently, we analyzed the drivers of regeneration success. First, we transformed all continuous covariates to their z-score. Next, we analyzed the correlation structure of covariates. As VPD_max_ is based on *T*_max_ and air humidity, VPD_max_ and *T*_max_ were highly correlated (*r* = 0.908). Thus, we omitted *T*_max_ in the multivariant analysis, assuming a stronger effect of drought than of heat on regenerating trees. The variance inflation factor (VIF) of all remaining continuous covariates was < 2, indicating a highly independent information value of each variable included in regression analysis (Dormann et al. 2013). Response variables were log-transformed to ensure a convergence of data to Gaussian distributions (Ives 2015). Subsequently, we tested the effects of VPD_max_, light level, deadwood presence and type, as well as browsing intensity on regeneration density, species diversity, and structural diversity, respectively, using multilevel Bayesian models. As browsing intensity could not be determined at two plots with missing regeneration, the number of plots analyzed was reduced to 43. Accounting for differences in environmental conditions and a diverging number of plots per site as well as spatial autocorrelation within sites, we allowed for variable intercepts by adding site as a random effect in the analysis. A Bayesian framework was used here because the incorporation of Markov Chain Monte Carlo (MCMC) sampling enables the analysis of complex data while not being restricted by the model’s degrees of freedom (Rossi et al. 2005). Bayesian models capture the full range of parameter uncertainty and allow the integration of prior information to restrict parameters to plausible ranges (McElreath 2016). We specified conservative (i.e., weakly informative) priors for all continuous covariates to restrict their initial parameter space to a reasonable range. The residuals of the final models were inspected for normality by QQ-plots. We used the Bayesian R^2^ as goodness-of-fit indicator. In addition, we predicted the posterior data distributions 50 times for a visual comparison between simulated and observed data distributions of response variables.

The R language and environment for statistical computing was employed for all analyses (R Development Core Team 2021). In particular, we harnessed the package tidyverse (Wickham 2019a) for data organization; rcompanion (Mangiafico 2021) for pairwise permutation tests; usdm (Naimi 2017) to test explanatory variables for multicollinearity; vegan (Oksanen et al. 2020) for NMDS ordination and ANOSIM; brms (Bürkner 2017) for Bayesian models and their evaluation; as well as ggplot2 (Wickham 2019b), bayestestR (Makowski et al. 2021), and bayesplot (Gabry et al. 2019) for visualizations.

## Results

### Treatment effects on microclimate and tree regeneration

**Fig. 4:**
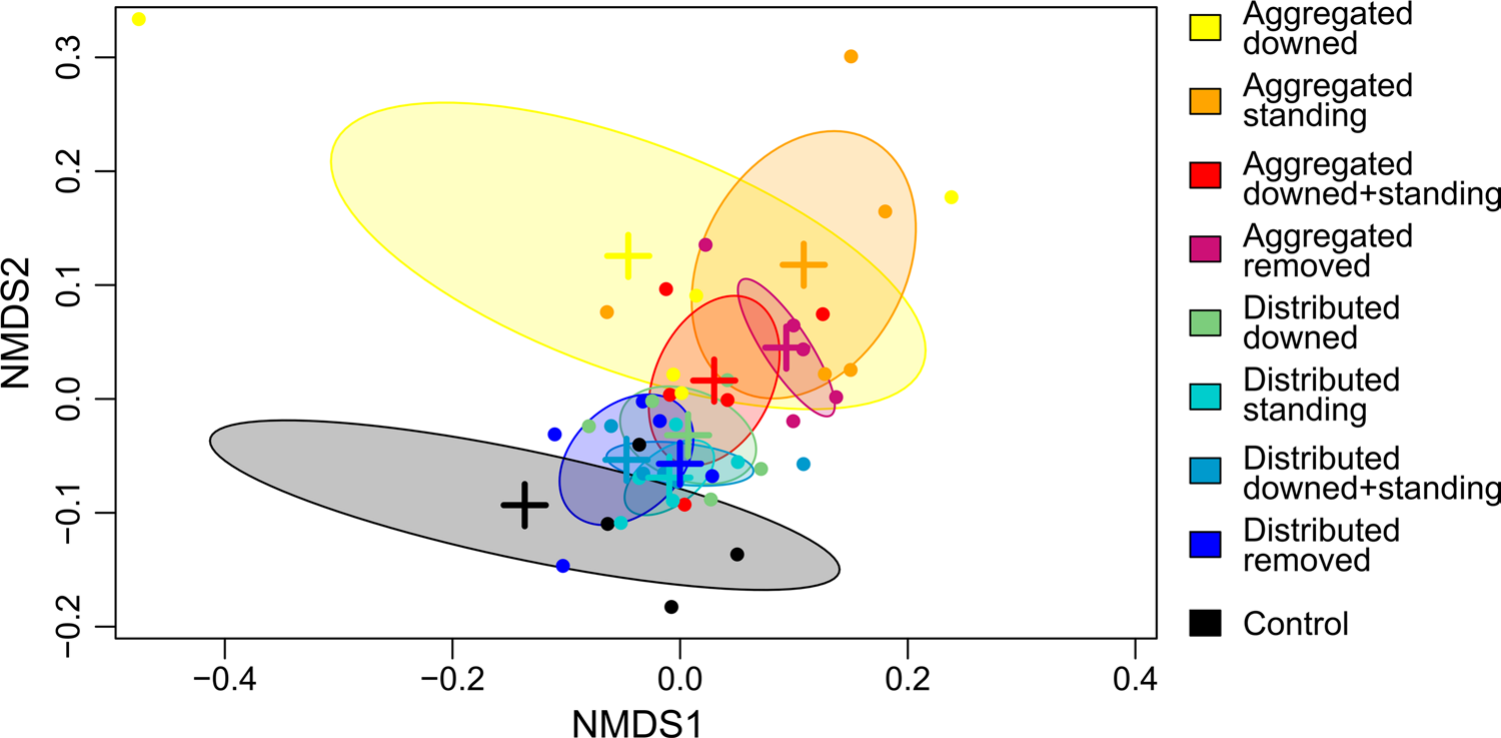
NMDS discriminating experimental treatments and untreated control plots. Presented are the first two axes of a three-dimensional ordination. Ellipses show the standard deviation of plots (points) around centroids (crosses). One control plot outlier was omitted in the visualization. Treatments are identified as aggregated/ distributed (referring to the spatial pattern of tree removal) and downed/ standing/ downed + standing/ removed (describing the treatment of deadwood)—see Fig. 2 for details

Microclimatic conditions and tree regeneration indicators were altered by treatments. Differences in treatment effects were primarily driven by the patterns of canopy tree removal (Fig. 4). The overall treatment effects of aggregated removal of canopy trees varied much stronger than those of distributed tree removal. Compared to distributed disturbance, aggregated disturbance filled a larger ordinal space as indicated by ellipses representing the standard deviation of plots in Fig. 4. In addition, the centroids of aggregated disturbances were further apart from each other than the centroids of distributed disturbances. Both the ellipses and centroids thus suggest larger variability in the effects of aggregated tree removal treatments. The ANOSIM confirmed a significant difference among treatments (*p* = 0.002); however, the dissimilarity of treatments was overall unincisive (R statistic: 0.129).

In a more detailed analysis of individual indicators, we identified some distinct differences in microclimatic conditions and tree regeneration among treatments (Tables 2, 3). However, only differences in variables related to microclimate were statistically significant, while there was high variability in treatment effects on browsing intensity and tree regeneration. Most notably, understory light conditions differed significantly among all canopy disturbance treatments (Table 2). On average, light levels increased by 6.8% points and + 3.4% points in the aggregated and distributed treatments, respectively, compared to the untreated control. Besides light level, also *T*_max_ and VPD_max_ were highest in plots with aggregated canopy tree removal. In comparison with control plots, *T*_max_ and VPD_max_ were 1.9 °C and 0.35 kPa higher under aggregated disturbance. In plots with distributed disturbance, T_max_ and VPD_max_ were only 0.3 °C and 0.03 kPa higher than in control plots. Microclimatic differences were only significant between aggregated and distributed canopy disturbances, but not between deadwood treatments (Table 3).

**Table 2:** Differences in microclimate (2016–2020) and tree regeneration (2020) among canopy treatments

Category	Attribute	Unit	Description	Canopy disturbance		
				Control (n = 5)	Distributed (n = 20)	Aggregated (n = 20)
Microclimate	T_max_	°C	Average diurnal maximum temperature during summer (JJA)	22.4 (1.4)^ab^	22.7 (1.3)^a^	24.3 (1.9)^b^
	VPD_max_	kPa	Average diurnal maximum vapor pressure deficit during summer (JJA)	1.13 (0.15)^ab^	1.16 (0.14)^a^	1.48 (0.35)^b^
	Light level	%	Total Site Factor * 100	7.1 (1.3)^a^	10.5 (2.1)^b^	13.9 (2.3)^c^
Regeneration	Browsing	%	Proportion of seedlings browsed	39.4 (40.5)^a^	45.8 (31.3)^a^	55.4 (28.9)^a^
	Regeneration density	n	Total number of seedlings per plot (sample area: 401.9 m^2^)	191 (227)^a^	254 (712)^a^	110 (173)^a^
	Species diversity	n	Effective number of seedling species	1.12 (0.75)^a^	1.73 (0.65)^a^	2.23 (0.93)^a^
	Structural diversity	n	Effective number of seedling height classes	1.52 (1.14)^a^	2.22 (0.69)^a^	2.30 (0.77)^a^

Presented are means and standard deviations (in parentheses) across all plots and treatments per canopy disturbance type. Letters indicate significant differences among treatments based on pairwise independence tests (α = 0.05)

**Table 3:** Differences in microclimate (2016–2020) and tree regeneration (2020) among deadwood treatments

Category	Attribute	Unit	Deadwood treatment				
			Control (n = 5)	Removed (n = 10)	Downed (n = 10)	Standing (n = 10)	Downed + Standing (n = 10)
Microclimate	T_max_	°C	22.4 (1.4)^a^	23.4 (1.5)^a^	23.5 (1.8)^a^	24.1 (2.5)^a^	23.0 (1.3)^a^
	VPD_max_	kPa	1.13 (0.15)^a^	1.30 (0.23)^a^	1.32 (0.27)^a^	1.44 (0.47)^a^	1.23 (0.21)^a^
	Light level	%	7.1 (1.3)^a^	12.0 (2.6)^b^	13.0 (3.2)^b^	12.3 (3.1)^b^	11.5 (2.4)^b^
Regeneration	Browsing	%	39.4 (40.5)^a^	46.3 (33.1)^a^	50.2 (31.5)^a^	44.8 (34.7)^a^	60.7 (22.1)^a^
	Regeneration density	n	191 (227)^a^	67 (87)^a^	418 (1003)^a^	155 (220)^a^	90 (90)^a^
	Species diversity	n	1.12 (0.75)^a^	1.98 (1.11)^a^	1.90 (0.96)^a^	1.99 (0.63)^a^	2.04 (0.67)^a^
	Structural diversity	n	1.52 (1.14)^a^	2.17 (0.69)^a^	2.02 (0.99)^a^	2.35 (0.56)^a^	2.50 (0.59)^a^

Presented are means and standard deviations (in parentheses) across all plots and treatments in the respective deadwood treatment category. Letters indicate significant differences among treatments based on pairwise independence tests (α = 0.05). See Table 2 for variable descriptions

The variation in canopy disturbance impacts on regeneration in 2020 was even larger than the variation in microclimate, thus leading to non-significant treatment effects (Table 2). The total number of seedlings in 2020 was 8,251. Eleven tree species were found in the regeneration layer, with European beech and Norway spruce being most prominent (i.e., 71.6% and 23.5%, respectively) (Table S2). On average, regeneration density was lowest on sites with aggregated disturbance. In contrast, the effective number of tree species was considerably higher in plots with aggregated disturbance (i.e., + 0.50 and + 1.11 species compared to distributed disturbance and control plots, respectively). Structural diversity of the regeneration layer was also higher in plots with aggregated (+ 0.78 height classes) and distributed disturbance (+ 0.70 height classes) compared to control plots. On average, more trees were browsed in plots with aggregated disturbance compared to distributed disturbance (+ 16.0%) and control plots (+ 9.6%). Browsing pressure was remarkably high in all height classes and for most species. On average across all plots, browsing pressure was slightly lower for trees at a height of 20–30 cm (54.2% browsed), while trees in the other two height classes were similarly affected by browsing (66.5% and 65.7% browsed in height classes 31–50 cm and > 50 cm, respectively). Among all species with a proportion of at least 1% in the regeneration layer, European beech and rowan (*Sorbus aucuparia* L.) were affected most strongly by browsing (both 74.4% browsed) (Table S2). In contrast, browsing pressure on Norway spruce was only moderate (30.0% browsed).

Deadwood treatments did not result in significant differences of the investigated indicators (Table 3). With regard to the average regeneration density per plot, the greatest difference among treatments was between retaining downed deadwood (up to 6.2 times more seedlings) and other treatments (Table 3). Furthermore, differences in species diversity were small between deadwood treatments. Browsing intensity and structural diversity were overall highest where both downed and standing deadwood remained after treatments, and lowest in control plots.

**Fig. 5:**
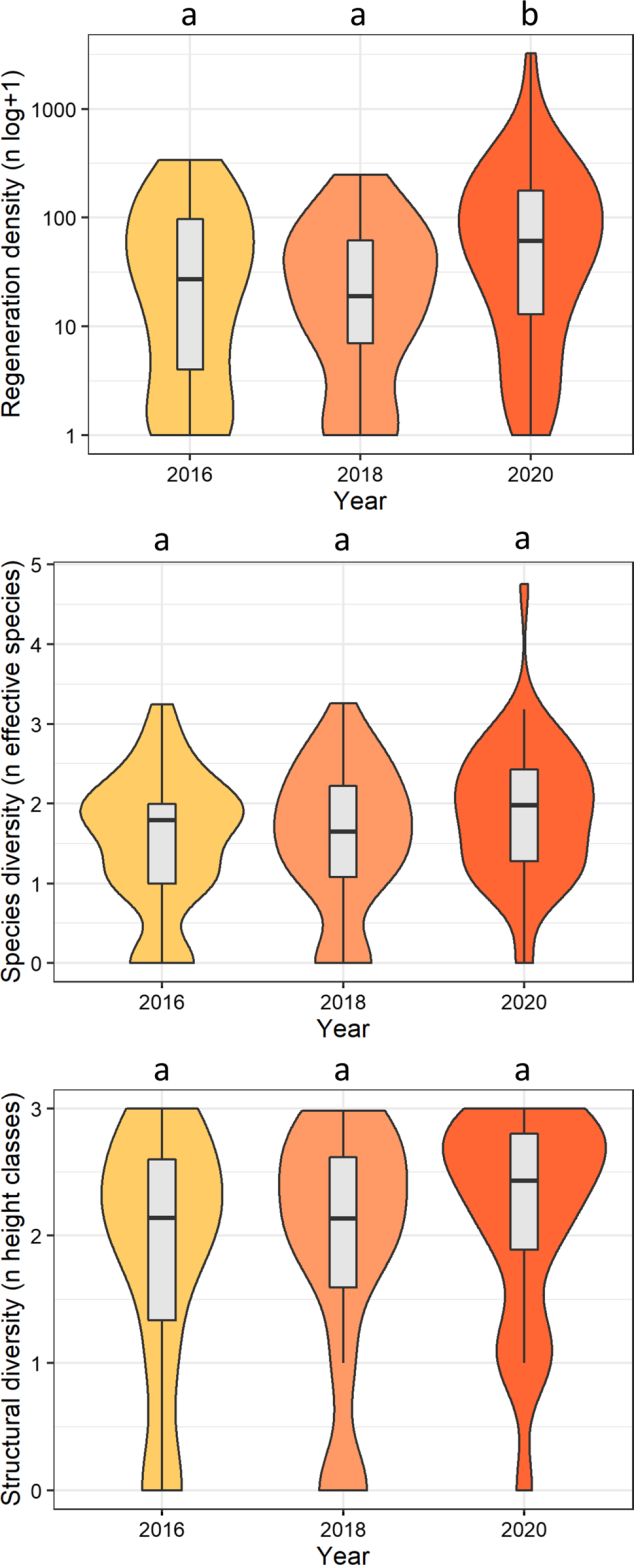
Temporal development of tree regeneration. Note that regeneration density (upper panel) is log-transformed. Letters indicate significant differences among years

On average, the forest canopy continued to open over time in both treated plots and control plots (Fig. S3). Comparing the years 2016 (immediately after treatment) and 2020, the greatest change was observed in plots with aggregated disturbance, where light increased by 6.2% points over time, followed by control plots (4.2% points), and plots with distributed disturbance (3.1% points). Regeneration density, species diversity, and structural diversity did not change notably between 2016 and 2018, but increased between 2018 and 2020 (Fig. 5). However, the only statistically significant increase between 2018 and 2020 was found for regeneration density. Across all canopy disturbance treatments, temporal patterns were similar for the two most common species, European beech and Norway spruce, with regeneration densities being highest in 2020 (Fig. S4). Differences among years were significant between 2018 and 2020, but not between 2016 and 2018. While the temporal development of Norway spruce regeneration density was independent of disturbance and deadwood treatments, we observed a strong increase in the number of European beech seedlings in plots with distributed disturbance (Fig. S5) and downed deadwood (Fig. S6) in the year 2020. Also, the regeneration density within the greatest height class culminated in 2020, and significant differences were only found between 2018 and 2020 (Fig. S7).

### Drivers of tree regeneration

**Fig. 6:**
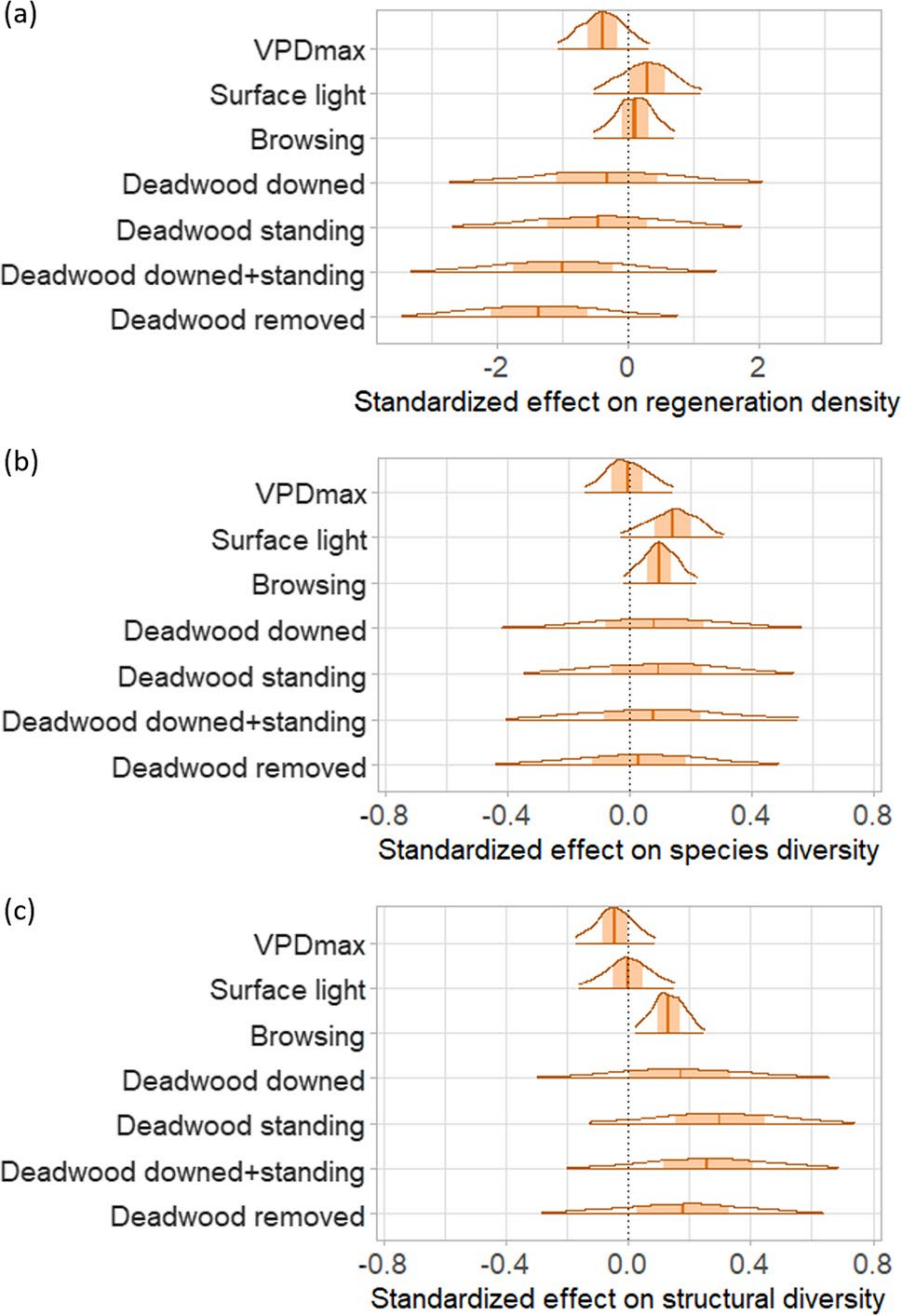
Standardized effects on (**a**) regeneration density, (**b**) species diversity, and (**c**) structural diversity. Presented are posterior distributions of population-level effects (i.e., regression coefficients) based on multilevel Bayesian models. Standardized effects indicate how much each response variable (log-transformed) changes in response to a change in the respective explanatory variable by one standard deviation. Visualized are the average population-level effects (vertical line), the 50% probability range (shaded area), and the 95% probability range (horizontal line). Note that the x-axis of (**a**) differs from (**b**) and (**c**)

Drivers of regeneration density, species diversity, and structural diversity differed among response variables (Fig. 6, S8–S10). However, all results exhibited high uncertainty. Light levels had the strongest positive effect on regeneration density and species diversity, but did not distinctly influence structural diversity. In contrast, VPD_max_ had a negative influence on regeneration density and structural diversity, but did not affect species diversity. While browsing did not affect regeneration density, it was positively associated with both species diversity and structural diversity. Deadwood treatment effects were highly uncertain and differed markedly among response variables, but the effects were similar across different types of deadwood (standing vs. downed). Overall, deadwood had a negative effect on regeneration density and a positive impact on species diversity and structural diversity.

The performance of our multilevel Bayesian models was overall sufficient. The variance explained was 40.7%, 49.2%, and 40.0%, for regeneration density, species diversity, and structural diversity models, respectively. Residuals were approximately normally distributed (Fig. S11). Predicted and observed data distributions were similar, indicating that our models were overall able to reproduce regeneration indicators based on the selected explanatory variables (Fig. S12).

## Discussion

### Tree regeneration: knowns and unknowns

We here studied tree regeneration in a replicated experiment, which has the advantage that a number of important drivers of tree regeneration (e.g., disturbance severity, disturbance pattern) can be controlled, increasing the inferential potential with regard to other explanatory variables. The disadvantage of experimental approaches such as applied here, however, is the inherently limited number of replicates/ observations. This makes identifying effects at common levels of statistical significance very challenging for highly variable processes such as tree regeneration. While our experiment revealed some distinct trends with regard to the implemented treatments, it also indicates high uncertainty in responses, as evidenced by high p-values and wide credibility intervals. Thus, our results indicate low predictability of treatment impacts on regeneration. Moreover, while the timing of our experiment enabled the investigation of microclimate effects on seedlings after a three-year dry and warm period, four vegetation periods after treatment are a limited amount of time for regeneration growth. Changes in regeneration dynamics over extended time frames are possible and require a continuation of inventories.

Notwithstanding high uncertainties, the results partially supported our hypotheses. We detected an overall positive impact of light on regeneration density (H1) and observed higher species diversity (H2) and structural diversity (H3) in aggregated compared to distributed disturbances and undisturbed plots. Furthermore, we found a negative relationship between the drying capacity of the air (VPD_max_) and regeneration density (H4), and an overall positive association between deadwood and species diversity as well as structural diversity (H6). In contrast, we neither anticipated the negative relationship between deadwood and regeneration density (H6), nor the positive correlation between browsing and species diversity as well as between browsing and structural diversity (H5).

As microclimate reflects the local environmental conditions, it is a better predictor for the regeneration niche than macroclimate (Lembrechts et al. 2019). We showed that aggregated disturbances allow more light to penetrate the canopy than distributed disturbances of the same severity (+ 3.4% points) (Table 2). At the same time, *T*_max_ and VPD_max_ were 1.6 °C and 0.32 kPa higher in aggregated than distributed disturbances (see also Kovács et al. 2020; Thom et al. 2020). Our study revealed that light conditions and the drying capacity of the air were of similar importance for regeneration success during the warm and dry years of our experiment, albeit with opposing effects (Fig. 6). As expected, light was overall positively associated with regeneration density and also increased species diversity. Light is an essential driver of photosynthesis (Wagner et al. 2009). Medium-sized gaps, such as those created in the aggregated disturbance treatment here (Table 2), provide heterogeneous light conditions, thus supporting tree species with diverging shade tolerances (Niinemets and Valladares 2006). In contrast, VPD_max_ reduced regeneration density and did not affect species diversity. Dry air induces evaporative water loss and reduces photosynthesis due to stomatal closure, inducing growth reductions or even causing mortality, especially if species are sensitive to drought (Williams et al. 2013). In addition, soils are drying quicker in larger gaps, reducing the ability of the shallow-rooting regeneration layer to take up water (Von Arx et al. 2013). Our analyses suggest that under the extreme climatic conditions after initialization of our experiment, the positive effect of increasing light and the negative effect of increasing VPD_max_ compensate each other, which could contribute to the rather small temporal changes in regeneration density (Fig. 5).

Our findings of a positive relationship between browsing and diversity in species and structures and no effect of browsing on regeneration density are somewhat unexpected, particularly considering the high browsing intensity observed on our plots. This result is challenged by a number of studies suggesting negative impacts of browsing on regeneration (Ammer 1996; Gill and Beardall 2001; Schulze et al. 2014; Reed et al. 2021). It is important to note that the relationships found here do not necessarily imply a causal effect of browsing. In effect, higher species diversity in the regeneration layer might attract ungulates and thus could result in increasing browsing levels with species diversity (Ohse et al. 2017; Borowski et al. 2021). Theoretically, a positive impact of browsing on species diversity could be explained, if browsing concentrates on the most dominant species (Wohlgemuth et al. 2002). However, this hypothesis is not supported by the browsing intensity by species observed in our study (Table S2). Instead, differences in seedling diversity might be partly explained by the overall low diversity of surrounding mature trees, that is, local differences might be driven by seed sources. Although the sites of our experiment were similar, local differences in baseline conditions cannot be entirely canceled by our study design. Furthermore, other studies have found a preference of ungulates for rare species (Ammer 1996; Gill and Beardall 2001; Schulze et al. 2014), while our study provides only little support for a higher browsing rate of rare species (Table S2). It is possible that the high browsing pressure across all sites repressed regeneration development acting as a homogenizing filter. In effect, canopy openings may lose their preeminent importance for regeneration performance if ungulate density exceeds a certain threshold (Horsley et al. 2003).

The low regeneration density in aggregated disturbance treatments might also be explained by high browsing pressure. Aggregated disturbances were characterized by the highest proportion of browsed seedlings and the lowest number of individuals as compared to the two other treatments (i.e., distributed disturbances and control). A study from eastern Poland found ungulate occurrence to be approximately twice as high in gaps compared to undisturbed forests (Kuijper et al. 2009). A possible explanation is provided by Hartley et al. (1997) who suggested that ungulates prefer a branching pattern of regenerating trees that emerges under improved light conditions (see also Brüllhardt et al. 2021). In the context of our study, it is also possible that abundant ungulates have prevented seedlings from reaching a height of 20 cm in aggregated disturbances and were thus not recorded by our inventories. Overall, the interactive effects between tree regeneration and browsing cannot be disentangled conclusively from our experiment, requiring further investigations e.g., using fencing treatments (Ammer 1996).

Our expectation of a positive deadwood effect on tree regeneration was confirmed for both species diversity and structural diversity. In contrast, regeneration density was overall negatively correlated with deadwood. Yet, uncertainty in the effects of deadwood on regeneration was very large, indicating that the number of plots per deadwood treatment (*n* = 10) was not sufficient to identify a distinct signal. Downed deadwood might support seedlings by creating a favorable microclimate very close to logs (Seibold et al. 2015; Marangon et al. 2022) or by preserving them from browsing (Hagge et al. 2019). Yet, our study cannot conclusively confirm these potential effects of deadwood on tree regeneration.

The high variability in treatment effects detected in our study is also the result of high complexity in processes driving tree regeneration (Seidl and Turner 2022). Consequently, high uncertainty in regeneration responses has likewise been reported for other forest types, such as sessile oak-hornbeam forests in Hungary (Tinya et al. 2020). For the forests of their study, no significant differences in regeneration density or species diversity could be identified among a set of standardized treatments. This reflects the complexity of regeneration processes that cannot solely be explained by the size of canopy openings or variation in deadwood, at least, within the first years after a disturbance. Also, Gottesman and Keeton (2017) found very high spatial variability in regeneration responses to stand structure and competition in northern hardwood forests in the USA. The critical role of microsites in shaping spatial variability in regeneration patterns has also been stressed in other studies. For instance, it was found that gap size controls not only light conditions, but also determines the number of seeds of different species on the forest floor within gaps that ultimately have the chance to establish a new generation of trees (Gray and Spies 1996; Kern et al. 2013). In particular, the availability of heavy seeds dispersed by autochory and zoochory often decreases strongly with gap size (Vittoz and Engler 2007). This also applies to the seeds of European beech. Mihók et al. (2005) found a negative influence of dispersal limitation on beech seedling establishment inside gaps of only 0.1 ha. Although the aggregated disturbances created here were relatively small (0.0625 ha), a reduced seed rain might partly explain the lower number of beech trees in aggregated disturbances (Fig. S5).

While our study has focused on microclimate effects on tree regeneration, other potentially important covariates have not been considered here. For instance, belowground resources, including water and nutrient availability, might be more important than light conditions for European beech regeneration during the first years after germination (Ammer et al. 2008). Moreover, processes such as masting, seed predation, competition with vascular plants other than trees, and herbivory of animals other than ungulates (e.g., small mammals) are important drivers of tree regeneration (Diaci et al. 2012; Zwolak et al. 2016).

### Management implications

Our study allows the derivation of several management implications. Aggregated canopy openings alter microclimatic conditions significantly more than distributed disturbances (Table 2). Although light was on average only 3.4% points higher, *T*_max_ and VPD_max_ were considerably elevated in the center of aggregated compared to distributed disturbances. As regeneration requires light but is susceptible to heat and drought extremes, harvest interventions aimed at initiating regeneration will have to find the right balance between increasing light levels and protecting regeneration from increasing weather extremes. Despite considerable differences in microclimatic conditions, we could not identify statistically significant differences in regeneration density or diversity among treatments (Table 2). We even recorded an increase in seedling numbers across all treatments in 2020, that is in the third year after the drought episode started in 2018 (Fig. 5, Figs. S4–S7). Conclusively, gaps of 625m^2^ (i.e., a disturbance size similar to typical group-selection cuts in Central Europe) might not be large enough to endanger regeneration of beech-dominated forests even under drought. In Europe, beech is predominantly managed by a regime of regular thinnings followed by a shelterwood cut initiating regeneration (Wagner et al. 2010). This management regime has supported regional beech dominance (Brunet et al. 2010; Wagner et al. 2010), given the high shade tolerance of the species. Our study indicates that irregular cuts (cf. our aggregated disturbance treatment) could promote tree diversity over classical, regular shelterwood cuts (cf. our distributed disturbance treatment), and thus help to increase the response diversity and resilience to climate change (Mori et al. 2013; Silva Pedro et al. 2015). Moreover, medium-sized canopy openings in typical irregular gap cuts might foster tree species better adapted to future environmental conditions (Stevens et al. 2015) and might increase the share of early-seral species, which generally have broader climatic niches (Swanson et al. 2011).

However, the significant effect of aggregated disturbances on microclimate in combination with lower seedling numbers indicates that future management actions have to be exercised with great caution in order not to exceed a threshold beyond which more extreme microclimates will no longer support tree regeneration. In particular, large canopy openings might not be well-suited to regenerate future beech-dominated forests because of the harsher microclimatic conditions, if drought intervals become shorter and their intensity increases (Qie et al. 2019). We showed that spatial patterns of management interventions matter, i.e., extracting the same amount of timber in a different spatial pattern, can have diverging impacts on forest microclimate. Our results indicate that it is possible to extract timber from mesic forests in group selection cuts without greatly endangering the microclimate buffering capacity against extreme weather events. Future work should focus on different gap sizes and extraction rates and also address how local site conditions (e.g., relief, topography) modify the interactions between canopy openings and microclimate.

Assuming that weather conditions will become increasingly challenging for tree regeneration, managing additional stressors such as browsing could play an even bigger role in the future than it does today. We have observed a very high browsing pressure in our study sites, which is common in many regions of Central Europe (Schulze et al. 2014). A reduction in browsing pressure could compensate some of the potential negative consequences of climate change on regeneration density. This holds particularly true in aggregated disturbances that not only exhibited the most extreme microclimate, but also the highest browsing pressure in our study. We thus call for more holistic ecosystem management approaches to safeguard future forest regeneration in order to sustain the provisioning of ecosystem services and biodiversity in a changing world.

## Supplementary Information

Below is the link to the electronic supplementary material.

Supplementary file 1.

## Data Availability

The data of this study can be obtained from Table S1.
