# Supplementary material for: Regeneration in European beech forests after drought: the effects of microclimate, deadwood and browsing
Source: Eur J For Res. 2022 Dec 8;142(2):259–73. doi: 10.1007/s10342-022-01520-1 (PMC10085954; doi:10.1007/s10342-022-01520-1)
Supplement: Supplementary file 1 [file 10342_2022_1520_MOESM1_ESM.docx]

**Supplement**

Table S1: Plot-level data used for the statistical analyses of this study.

| Site | Disturbance pattern | Deadwood | Tmax (°C) | VPDmax (kPa) | Light level (%) | Browsing (%) | Regeneration density (n) | Species diversity (n) | Structural diversity (n) |
| --- | --- | --- | --- | --- | --- | --- | --- | --- | --- |
| GUG | aggregated | downed+standing | 22.89 | 1.28 | 20.45 | 37.5 | 8 | 2.46 | 2.46 |
| GUG | aggregated | downed | 23.36 | 1.38 | 19.35 | NA | 0 | 0 | 0 |
| GUG | aggregated | removed | 24.63 | 1.48 | 16.5 | 74.07 | 27 | 4.76 | 2.79 |
| GUG | aggregated | standing | 28.59 | 2.38 | 19.95 | 0 | 1 | 1 | 1 |
| GUG | control | control | 23.51 | 1.37 | 7.55 | 0 | 2 | 1 | 1 |
| GUG | distributed | downed+standing | 22.59 | 1.17 | 13.85 | 100 | 1 | 1 | 1 |
| GUG | distributed | downed | 22.63 | 1.16 | 11.77 | 0 | 3 | 1 | 1 |
| GUG | distributed | removed | 22.71 | 1.16 | 13.8 | 3.9 | 77 | 1.42 | 1.81 |
| GUG | distributed | standing | 22.48 | 1.19 | 8.82 | 13.51 | 37 | 1.28 | 2.19 |
| JMH | aggregated | downed+standing | 23.1 | 1.33 | 14.97 | 65.85 | 123 | 1.97 | 2.87 |
| JMH | aggregated | downed | 22.47 | 1.07 | 18.12 | 67.42 | 89 | 2 | 2.81 |
| JMH | aggregated | removed | 24.92 | 1.61 | 13.67 | 77.78 | 9 | 1.98 | 1.89 |
| JMH | aggregated | standing | 25.02 | 1.68 | 14.77 | 100 | 7 | 2.6 | 2.73 |
| JMH | control | control | 21.78 | 1.1 | 5.4 | 91.48 | 223 | 1.03 | 2.56 |
| JMH | distributed | downed+standing | 22.07 | 1.13 | 11.83 | 74.05 | 185 | 2.03 | 2.95 |
| JMH | distributed | downed | 22.58 | 1.2 | 13.12 | 93.55 | 3255 | 1.18 | 2 |
| JMH | distributed | removed | 21.94 | 1.16 | 11.45 | 0 | 1 | 1 | 1 |
| JMH | distributed | standing | 23.24 | 1.29 | 12.28 | 83.33 | 174 | 1.74 | 2.8 |
| KUH | aggregated | downed+standing | 21.69 | 1.01 | 17.65 | 85.42 | 48 | 2.62 | 2.61 |
| KUH | aggregated | downed | 23.22 | 1.21 | 21.58 | 56.92 | 65 | 3.19 | 2.88 |
| KUH | aggregated | removed | 24.38 | 1.54 | 17.78 | 80 | 15 | 2.64 | 2.64 |
| KUH | aggregated | standing | 21.58 | 1.08 | 20.62 | 39.69 | 320 | 2.18 | 1.97 |
| KUH | control | control | 21.53 | 1.05 | 9.43 | 16.01 | 556 | 2.04 | 2.76 |
| KUH | distributed | downed+standing | 22.39 | 1.06 | 11.68 | 47.73 | 264 | 2.81 | 2.87 |
| KUH | distributed | downed | 22.12 | 1.12 | 15.78 | 46.29 | 283 | 2.33 | 2.92 |
| KUH | distributed | removed | 22.79 | 1.11 | 13.6 | 29.96 | 247 | 2.29 | 2.14 |
| KUH | distributed | standing | 22.79 | 1.25 | 12.43 | 25.19 | 135 | 2.61 | 2.54 |
| TUM | aggregated | downed+standing | 23.47 | 1.11 | 7.4 | 28.3 | 53 | 1.56 | 2.62 |
| TUM | aggregated | downed | 27.83 | 2.01 | 22.02 | 86.27 | 335 | 2.23 | 2.99 |
| TUM | aggregated | removed | 24.72 | 1.37 | 13.75 | 76.24 | 202 | 1.8 | 2.38 |
| TUM | aggregated | standing | 26.24 | 1.52 | 13.18 | 14.37 | 717 | 1.98 | 2.98 |
| TUM | control | control | 24.14 | 1.16 | 5.35 | 50 | 176 | 1.52 | 1.28 |
| TUM | distributed | downed+standing | 25.32 | 1.46 | 9.65 | 44.51 | 164 | 1.08 | 2.96 |
| TUM | distributed | downed | 25.32 | 1.38 | 6.38 | 12.82 | 78 | 1.72 | 2.03 |
| TUM | distributed | removed | 23.89 | 1.12 | 10.48 | 46.51 | 43 | 1.44 | 2.84 |
| TUM | distributed | standing | 23.53 | 1.04 | 8.45 | 26.67 | 75 | 1.32 | 2.42 |
| TWF | aggregated | downed+standing | 24.91 | 1.69 | 16.92 | 60 | 20 | 2.85 | 2.45 |
| TWF | aggregated | downed | 22.57 | 1.29 | 14.38 | 30 | 60 | 2.42 | 2.23 |
| TWF | aggregated | removed | 23.79 | 1.48 | 21.88 | 8.33 | 48 | 1.51 | 1.19 |
| TWF | aggregated | standing | 26.66 | 2.03 | 19.65 | 65 | 60 | 2.83 | 2.43 |
| TWF | control | control | 20.82 | 0.97 | 6.05 | NA | 0 | 0 | 0 |
| TWF | distributed | downed+standing | 21.49 | 1.05 | 12.12 | 63.33 | 30 | 2 | 2.16 |
| TWF | distributed | downed | 23.28 | 1.34 | 14.32 | 58.33 | 12 | 2.94 | 1.33 |
| TWF | distributed | removed | 20.34 | 0.94 | 8.6 | 66.67 | 3 | 1 | 3 |
| TWF | distributed | standing | 20.38 | 0.92 | 11.47 | 80 | 20 | 2.36 | 2.43 |

Table S2: Regeneration density per species and browsing state.

| Species | Unbrowsed (n) | Browsed (n) | Browsed (%) |
| --- | --- | --- | --- |
| *Abies alba* | 2 | 15 | 88.2 |
| *Acer pseudoplatanus* | 1 | 5 | 83.3 |
| *Betula pendula* | 1 | 1 | 50 |
| *Fagus sylvatica* | 1515 | 4395 | 74.4 |
| *Fraxinus excelsior* | 1 | 1 | 50 |
| *Picea abies* | 1357 | 581 | 30 |
| *Pseudotsuga menziesii* | 1 | 0 | 0 |
| *Quercus robur* | 1 | 1 | 50 |
| *Rhamnus frangula* | 1 | 1 | 50 |
| *Salix spp.* | 1 | 5 | 83.3 |
| *Sorbus aucuparia* | 92 | 267 | 74.4 |
| *Larix spp.* | 0 | 1 | 100 |
| *Pinus spp* | 0 | 2 | 100 |
| *Populus spp.* | 0 | 3 | 100 |
| Total | 2973 | 5278 | 66.7 |


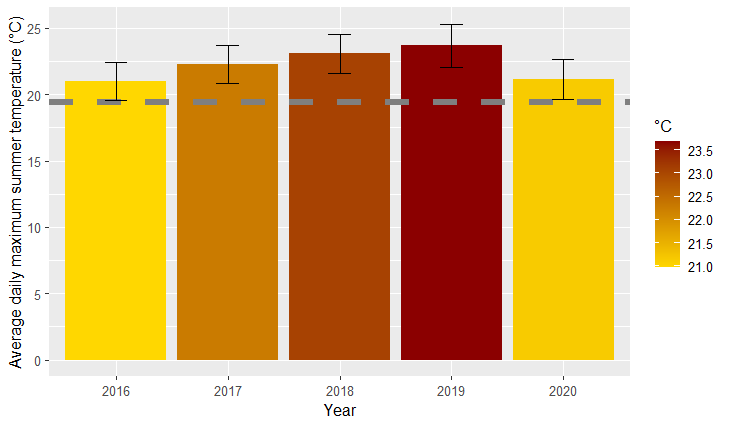


Figure S1: Average over the daily maximum summer temperature (T_max_) at the experimental sites. Each bar represents the average T_max_ across the five sites. Error bars show the standard deviation across sites. The dashed line indicates the 30-year average T_max_ of the period 1961 – 1990. Values were retrieved from 1 km grids of Germany's National Meteorological Service (Deutscher Wetterdienst), available at <https://opendata.dwd.de/climate_environment/CDC/grids_germany/> (accessed 09/17/2021).


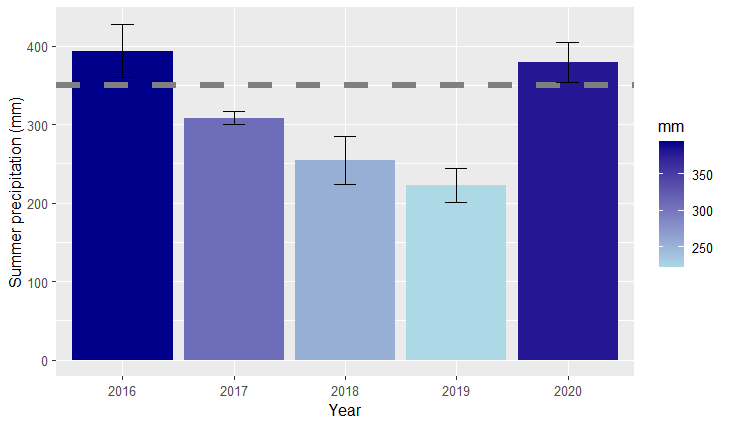


Figure S2: Total summer precipitation (P_sum_) at the experimental sites. Each bar represents the average P_sum_ across the five sites. Error bars show the standard deviation across sites. The dashed line indicates the 30-year average P_sum_ of the period 1961 – 1990. Values were retrieved from 1 km grids of Germany's National Meteorological Service (Deutscher Wetterdienst), available at <https://opendata.dwd.de/climate_environment/CDC/grids_germany/> (accessed 09/17/2021).


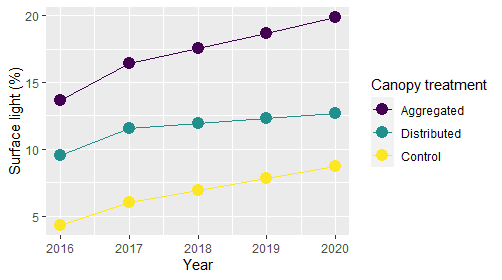


Figure S3: Change in light level over time. Presented are average light conditions at plots with an aggregated and distributed disturbance patterns as well as untreated control plots. Light level corresponds to the TSF. Note that the years 2018 and 2019 were interpolated.


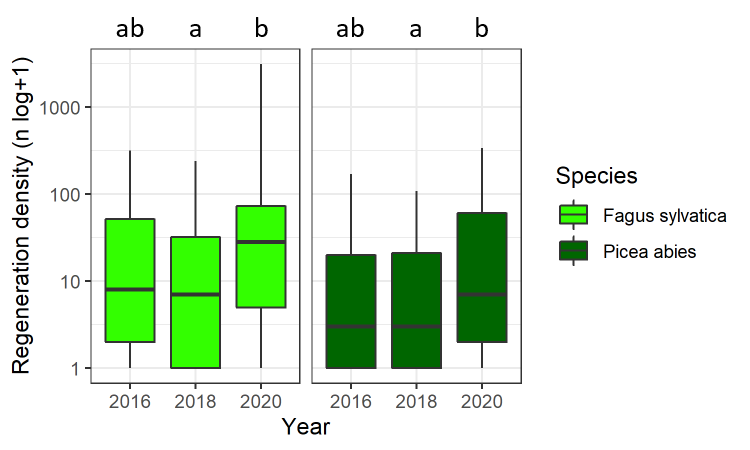


Figure S4: Temporal development of regeneration density for the two most common species *Fagus sylvatica* and *Picea abies*. Regeneration density refers to the total number of seedlings per plot (sample area: 401.9 m²). Letters indicate significant difference (α = 0.05).


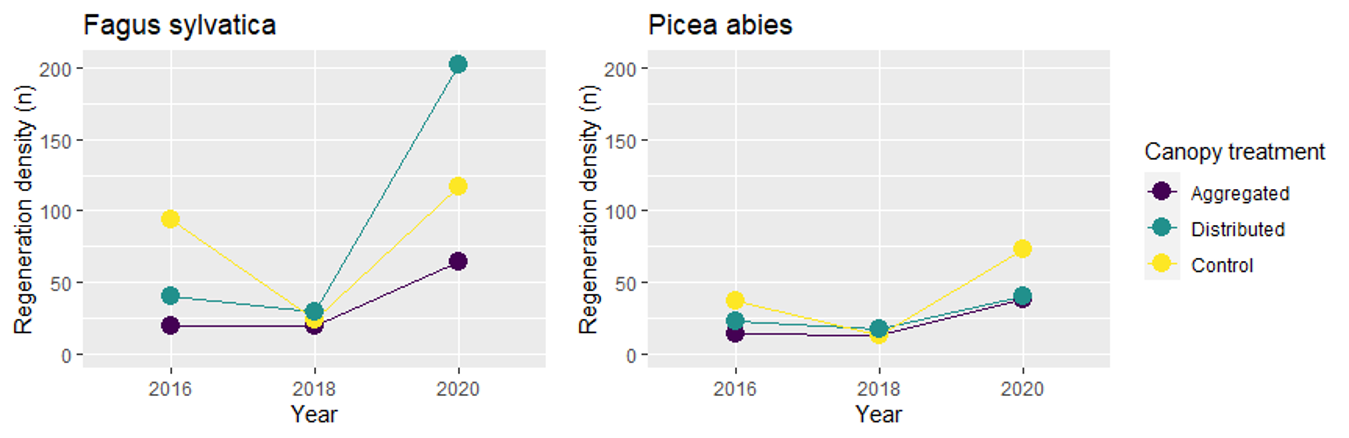


Figure S5: Temporal development of regeneration density for *Fagus sylvatica* and *Picea abies* for each canopy treatment (disturbance) pattern. Regeneration density refers to the total number of seedlings per plot (sample area: 401.9 m²).


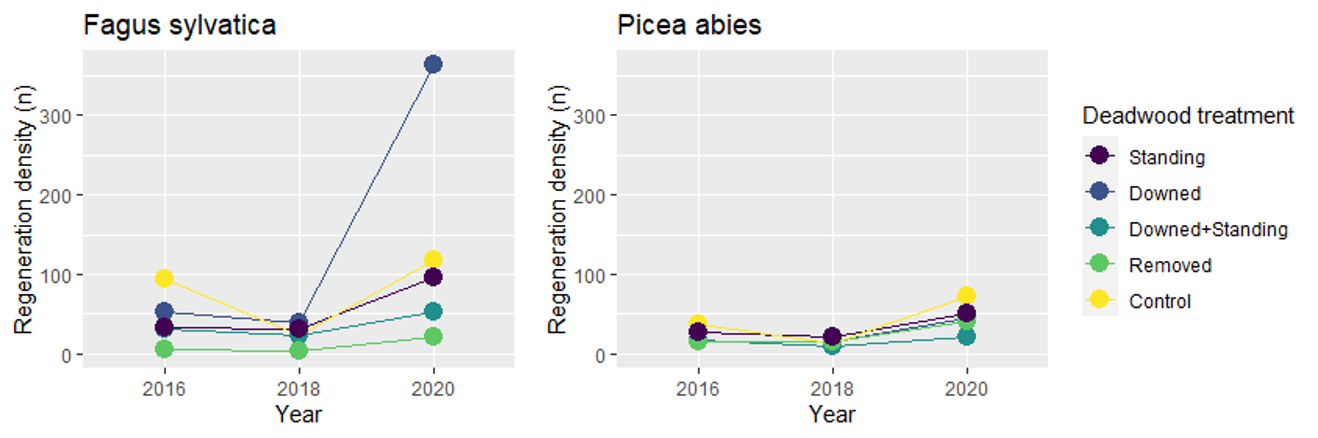


Figure S6: Temporal development of regeneration density for *Fagus sylvatica* and *Picea abies* for each deadwood treatment. Regeneration density refers to the total number of seedlings per plot (sample area: 401.9 m²).


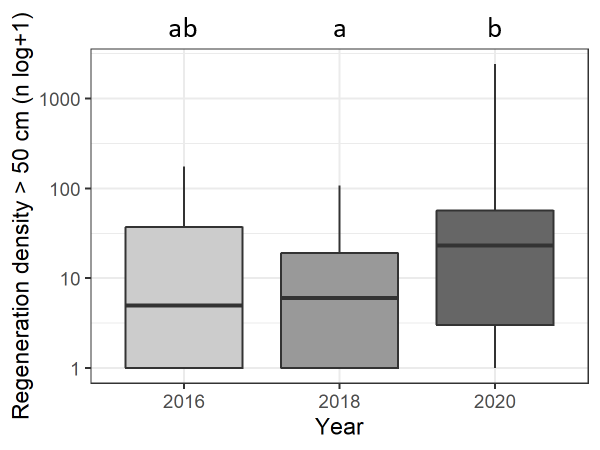


Figure S7: Temporal development of regeneration density for the greatest height class (> 50 cm). Regeneration density refers to the total number of seedlings per plot (sample area: 401.9 m²). Letters indicate significant difference (α = 0.05).


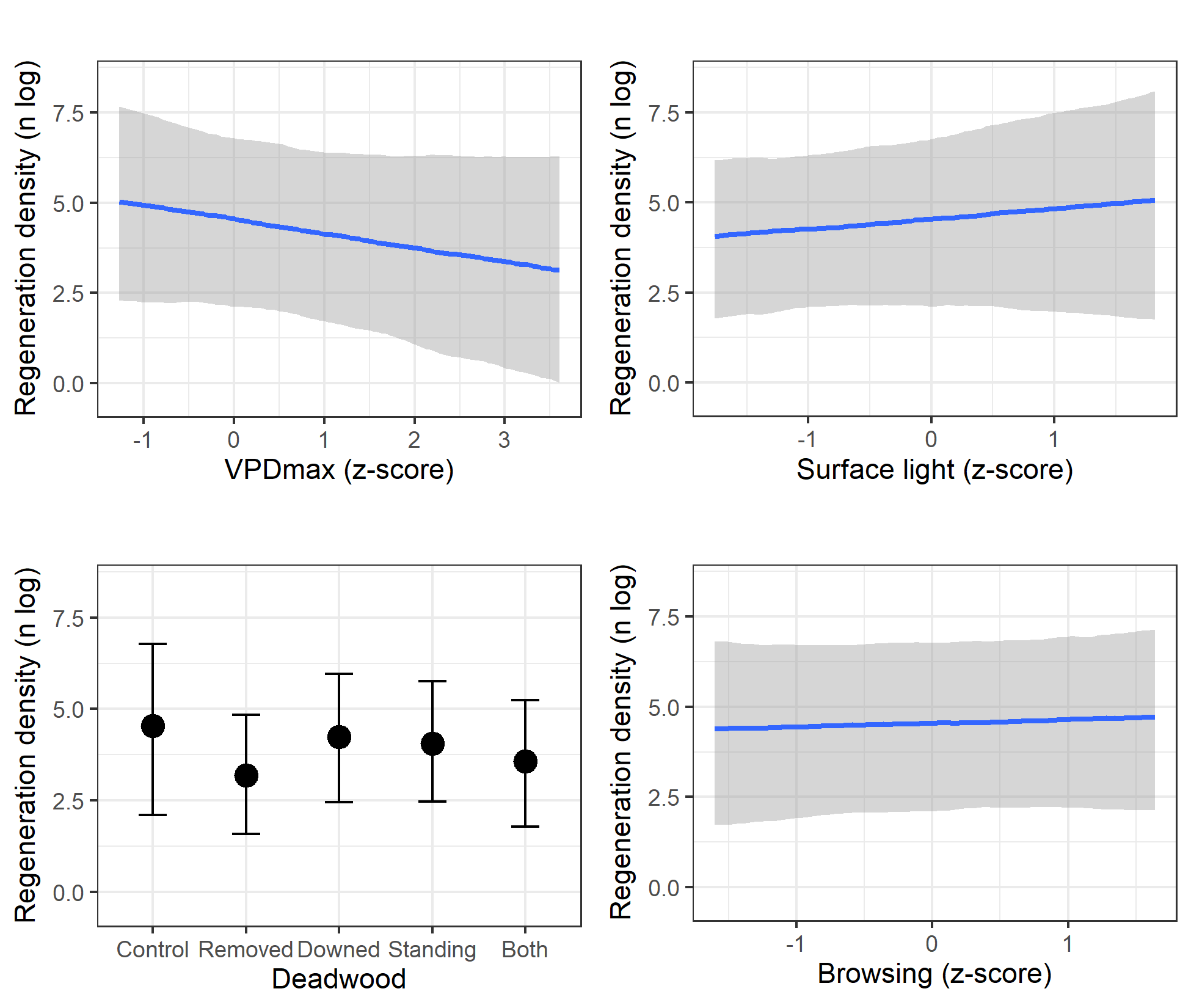


Figure S8: Standardized regression plots for variables explaining regeneration density. Regeneration density refers to the total number of seedlings per plot (sample area: 401.9 m²). Deadwood both = downed + standing deadwood.


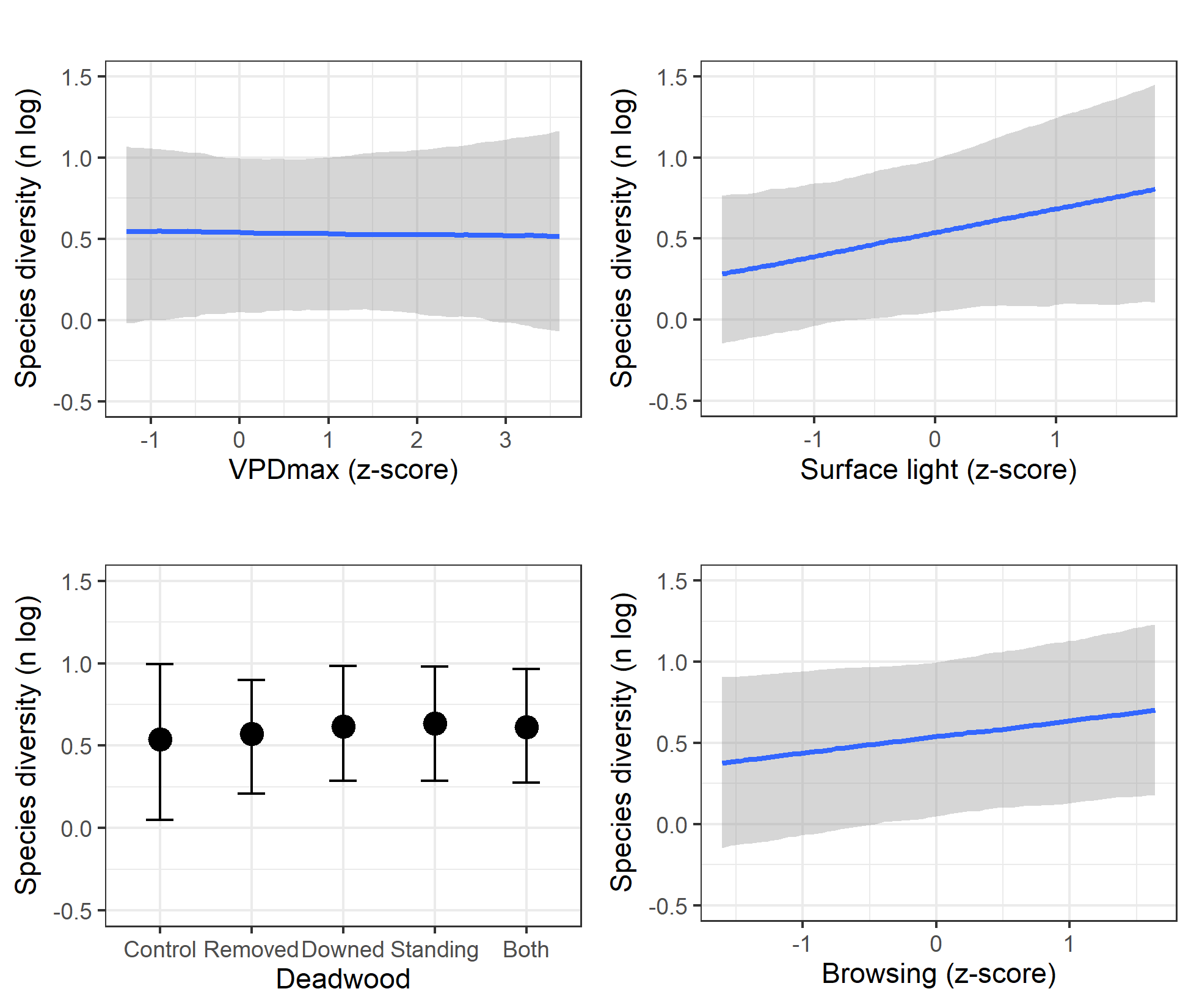


Figure S9: Standardized regression plots for variables explaining species diversity. Deadwood both = downed + standing deadwood.


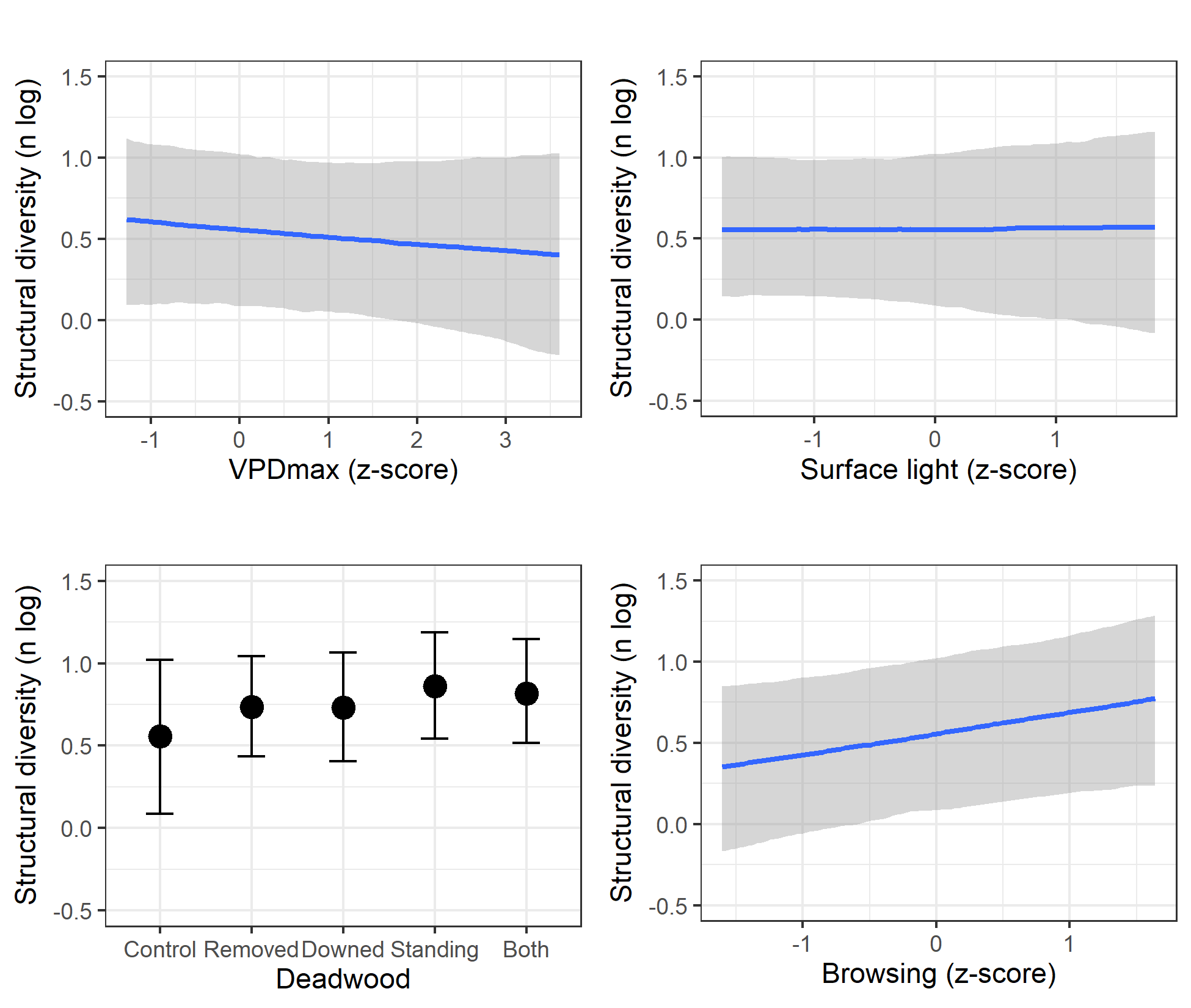


Figure S10: Standardized regression plots for variables explaining structural diversity. Deadwood both = downed + standing deadwood.


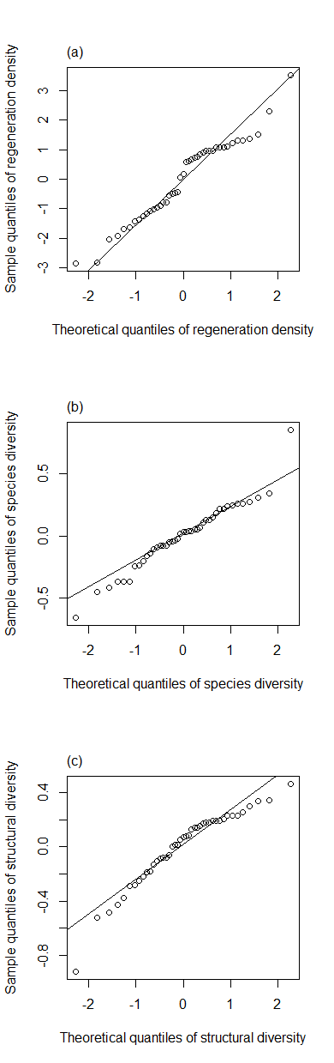


Figure S11: QQ-Plots of the Bayesian multilevel model residuals for (a) regeneration density, (b) species diversity, and (c) structural diversity. Regeneration density refers to the total number of seedlings per plot (sample area: 401.9 m²).


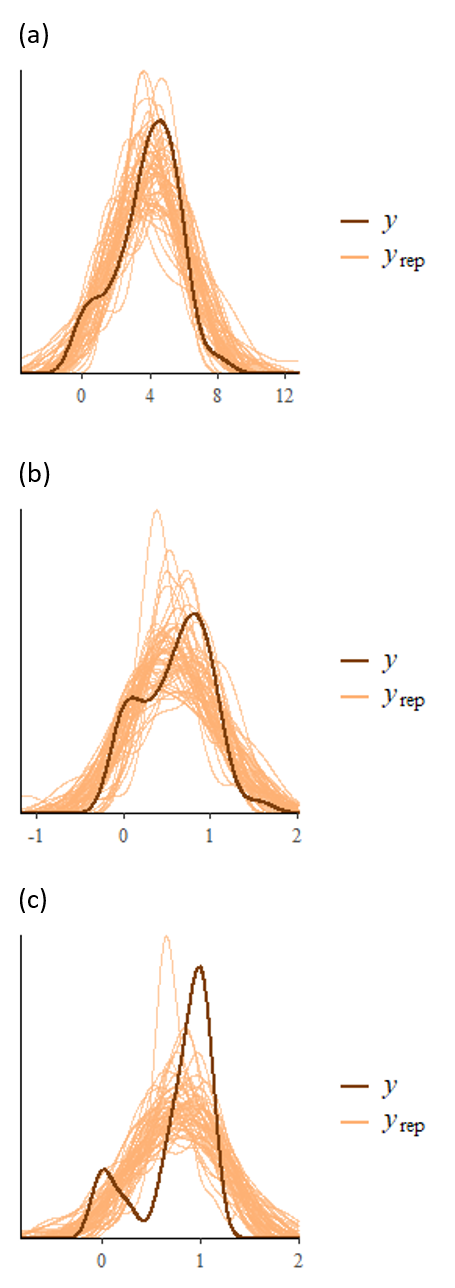


Figure S12: Posterior predictive distributions of the Bayesian multilevel models for (a) regeneration density, (b) species diversity, and (c) structural diversity. y indicates the observed data distribution while y_rep_ shows 50 simulated data distributions.
